# A desirability-based multi objective approach for the virtual screening discovery of broad-spectrum anti-gastric cancer agents

**DOI:** 10.1371/journal.pone.0192176

**Published:** 2018-02-08

**Authors:** Yunierkis Perez-Castillo, Aminael Sánchez-Rodríguez, Eduardo Tejera, Maykel Cruz-Monteagudo, Fernanda Borges, M. Natália D. S. Cordeiro, Huong Le-Thi-Thu, Hai Pham-The

**Affiliations:** 1 Escuela de Ciencias Físicas y Matemáticas, Universidad de Las Américas, Quito, Ecuador; 2 Departamento de Ciencias Biológicas, Universidad Técnica Particular de Loja, Loja, Ecuador; 3 Facultad de Ingenieria y Ciencias Agropecuarias, Universidad de Las Américas, Quito, Ecuador; 4 CIQUP/Departamento de Química e Bioquímica, Faculdade de Ciências, Universidade do Porto, Porto, Portugal; 5 REQUIMTE/Departamento de Química e Bioquímica, Faculdade de Ciências, Universidade do Porto, Porto, Portugal; 6 Department of General Education, West Coast University—Miami Campus, Doral, Florida, United States of America; 7 VNU School of Medicine and Pharmacy, Vietnam National University, Hanoi, Vietnam; 8 Hanoi University of Pharmacy, Hanoi, Vietnam; Istituto di Genetica Molecolare, ITALY

## Abstract

Gastric cancer is the third leading cause of cancer-related mortality worldwide and despite advances in prevention, diagnosis and therapy, it is still regarded as a global health concern. The efficacy of the therapies for gastric cancer is limited by a poor response to currently available therapeutic regimens. One of the reasons that may explain these poor clinical outcomes is the highly heterogeneous nature of this disease. In this sense, it is essential to discover new molecular agents capable of targeting various gastric cancer subtypes simultaneously. Here, we present a multi-objective approach for the ligand-based virtual screening discovery of chemical compounds simultaneously active against the gastric cancer cell lines AGS, NCI-N87 and SNU-1. The proposed approach relays in a novel methodology based on the development of ensemble models for the bioactivity prediction against each individual gastric cancer cell line. The methodology includes the aggregation of one ensemble per cell line using a desirability-based algorithm into virtual screening protocols. Our research leads to the proposal of a multi-targeted virtual screening protocol able to achieve high enrichment of known chemicals with anti-gastric cancer activity. Specifically, our results indicate that, using the proposed protocol, it is possible to retrieve almost 20 more times multi-targeted compounds in the first 1% of the ranked list than what is expected from a uniform distribution of the active ones in the virtual screening database. More importantly, the proposed protocol attains an outstanding initial enrichment of known multi-targeted anti-gastric cancer agents.

## Introduction

Gastric cancer (GC) is the third leading cause of cancer-related mortality worldwide. Despite advances in prevention, diagnosis and therapy, GC is still regarded as a global health concern. The high mortality of GC is mainly due to late diagnosis and poor response to the currently available therapeutic regimens [1]. Overall, clinical response to chemotherapy ranges from 20 to 40%. Fluoropyrimidine- and platinum-based chemotherapeutic treatments are recommended in the neoadjuvant or adjuvant setting as the first-line treatment in patients with advanced and unresectable GC. The search for alternatives to conventional chemotherapeutic regimens is an active field of work in drug-design related to GC. In this sense, a number of recent papers reports advances in the discovery of drug targets e.g. key components of specific oncogenic pathways that could be targeted by novel therapies. However, based on the results of phase III clinical trials, targeted therapies have shown to offer only a limited survival advantage of a few months (1.5–2.2 months) [2].

One of the reasons that may explain the poor clinical outcomes of GC therapy is the highly heterogeneous nature of this disease which occurs at both morphological and molecular levels. It is noteworthy that GC heterogeneity encompasses not only interpatient variability (intertumour heterogeneity), but also variations within the same tumor (intratumor heterogeneity). According to the Laurén’s classification [3], there are two main GC subtypes: intestinal and diffuse GC. Besides that, in 1995, Carneiro et al. [4] elucidated the biological features and prognostic significance of a third subtype known as mixed GC which is defined by a dual pattern of differentiation: glandular/solid (intestinal) and isolated-cell carcinoma (diffuse). The recent advent of Next Generation Sequencing Technologies e.g. RNA-seq, made it amenable to look for molecular signatures in different GC cell-lines. Based on gene expression signatures Lei et al. [5] identified three “molecular subtypes” that show differences in molecular/genomic features, morphology, “carcinogenic” pathways, and response to therapy that overlap only partially with the “morphological subtypes” described by Laurén and Carneiro.

In a scenario of morphological and molecular heterogeneity as the one observed in GC, more effective therapeutic regimens able to target the heterogeneous tumor cell populations are urgently needed. Single target-centered therapies might not be the best way to proceed and drug discovery for GC must then depart from the target-centered paradigm which by its very nature overlooks the complexity of cell and makes the process of validation of the drug targets uncertain [6]. It is imperative to search for alternatives like systemic approaches, where drugs are developed to interfere with multiple components of the pathological process. Multi-target drug design (MTDD) aims at identifying multi-targeted drugs to increase the probabilities of inducing the desired phenotype or therapeutic effect [7]. Multi-targeted drugs are prospectively and intentionally designed to specifically interact with multiple targets involved in a disease state. Clinically successful multi-targeted drugs have shown several benefits compared to their single-target relatives, such as improved safety profiles, and reduced resistance activities. Such benefits can be obtained by limiting unwanted cross-reactivities via optimization of target selectivity [8].

The rationale behind the efficiency of multi-targeting in drug discovery relies on the network-like nature of complex systems [9]. System biology analyses have shown little effect on disease networks after the deletion of individual protein nodes, suggesting that targeting multiple proteins is required to perturb robust disease phenotypes [10]. Resistance of GC cells to targeted therapies, in which only one molecule is perturbed at the time [2], might arise from the activation of compensating signaling loops. This has been reported for other cancer types when the inhibition of kinases by targeted drugs is evaded by compensatory changes in the signaling pathways [11,12].

The popularity of MTDD resides in the fact that the rational design of multi-target compounds is conceptually attractive [7]. The development and application of computational methods and tools for in silico drug discovery is a starting point in MTDD. Computational approaches include, but are not limited to, cheminformatics, molecular similarity, docking, molecular dynamics, virtual screening (VS) and Quantitative Structure-Activity Relationships (QSAR) [8]. Among these, QSAR has proven to be a very versatile technique for the creation of multi-target models.

For instance, QSAR can be used to generate models against multiple viral, bacterial and fungal species (see [8] and references therein) resulting in multi-species models. The former can be achieved by incorporating species-dependent molecular descriptors into the QSAR pipeline. Multi-species QSAR models can achieve high retrieval rates (72–85%) and moderately low false-fit rates (15–28%) [8]. More recently, multi-target QSAR models have been developed that incorporate data from several cell lines by a sort of multi-species strategy [13]. According to the authors of the study, the integration of experimental data from multiple cell lines into the QSAR pipeline provided several advantages compared to single cell line QSAR modeling. Among the advantages, authors mentioned that the use of data from multiple cell lines resulted in i) better handling the inherent noise of experimental activity measurements and ii) reducing the detrimental effects that arise from the relatively small number of training data that is commonly available for a single cell line [13].

The fact that QSAR could be successfully used to integrate pharmacological data from multiple cell lines becomes especially appealing in the context of GC although none of such models have been reported so far. As mentioned earlier, in the context of GC, multi cell lines QSAR models can be instrumental to derive the commonly important features of compounds active against all cell lines. This information can guide the design of therapies with lower failure due to resistance in specific cell lines (cancer subtypes).

To this end however, a proper VS strategy must be used to prioritize chemicals potentially active against all given cell lines. As demonstrated in our previous research [14], a QSAR-based classification scheme can be combined with an optimization process based on desirability functions to tackle the problem of compound prioritization. Since desirability-based functions are multi-objective by nature (see [14] and references therein for a review on the topic), they are ideal for MTDD and have been used side-by-side with QSAR studies [15–17]. On the other hand, as pointed out by QSAR modeling practitioners, ensemble modeling can be effectively employed for addressing problems where highly heterogeneous data are studied [14,18–23].

Given that, in the present work we explore the application of MTDD for the VS discovery of chemical compounds simultaneously active against the GC AGS, NCI-N87 and SNU-1 cell lines [24,25]. The corresponding bioactivity data were collected from the ChEMBL database [26]. The data sets were subjected to a thorough curation protocol and employed to train generalizable ensemble models per endpoint (bioactivity against a GC cell line). Part of the collected data with activity measurements against the three cell lines was reserved for VS validations. These validations also involved a set of decoy molecules to resemble a VS scenario. Finally, we evaluated the VS performance of various combinations of endpoints’ ensembles by employing a desirability-based approach. The whole modeling process was implemented in MATLAB [27].

## Computational methods

### Data sets and molecular descriptors

Compounds experimentally evaluated against the gastric cell lines AGS, NCI-N87 and SNU-1 [24,25] were retrieved from the ChEMBL database [26]. Anti-tumoral activity was reported as either EC50 (concentration inhibiting half of cell culture grow) or GI (percent of cell grow inhibition at a fixed concentration). Although data was collected from a single database (ChEMBL), bioactivity reports come from different experiments. This is a scenario common to most QSAR modeling applications which can negatively affects their performance. Considering that regression-based modeling is more sensitive to data heterogeneity, classification models were therefore developed in this research.

For classification modeling, samples were assigned to the actives or inactives classes (groups). Each group was coded as 1 and 0, respectively. Samples were considered to belong to the actives group if they were able to inhibit at least 50% of cell proliferation at a concentration equals to or lower than 10 μM. Compounds were considered inactives if they inhibited less than 50% of cellular proliferation at the former concentration. For GI determinations, only samples for which an unambiguous classification could be established were considered. This means that chemicals inhibiting less than 50% of cell proliferation at concentrations lower than 10 μM were removed from the data set. The same action was taken for compounds inhibiting more than 50% of cell proliferation at concentrations higher than 10 μM.

The chemical structures were coded in smiles format and then converted to SD files (SDF) using the ChemAxon’s JChem for Excel (6.3.1.1807) program [28]. Molecular protonation states were the same provided in ChEMBL. Each data set, as well as the decoy compounds (see next sections), were curated following the guidelines proposed in the literature [29,30]. The removal of explicit hydrogen atoms, the rings aromatization, the normalization of specific chemotypes such as nitro to one unique representation, the curation of tautomeric forms, the striping of salts and small fragments removal were performed with ChemAxon’s Standardizer [31]. Duplicate compounds were detected with the EdiSDF tool of the ISIDA/QSPR package [32].

Data sets were split into training, test and external sets employing the sphere exclusion algorithms previously reported [23]. The samples for the VS Validation Set (VSVS) were extracted using the Maximal-Minimal Dissimilarity Selection algorithm implemented on the ChemAxon’s JKlustor software [33]. Decoy molecules were selected employing the DUD-E server [34]. The external dataset was used only for challenging the model predictive power without playing as a decision maker at any step of the modeling process.

The ISIDA Fragmentor software was used to calculate 2D fragment descriptors [35,32]. The “sequences”, “atom centered fragments” and “triplets” types of fragments considering only sequences of atoms and bonds were computed. Only the shortest paths from one atom to the other were used. For each type of sequence, the minimal (nmin) and maximal (nmax) counts of constituent atoms were defined as nmin = 2 and nmax = 8.

The above described descriptors were calculated for the training dataset. Afterward, fragments returning nearly constant values (for 99%) of the samples were removed. The Minimal Redundancy Maximal Relevance (mRMR) algorithm [36] was applied to the reduced data set to keep only the top 250 fragments according to the Mutual Information Quotient (MIQ) score. The same subset of 250 fragments was then computed for the other data sets to be predicted later.

### Training base models

A total of 1001 base models were trained for each endpoint employing the training data set. To ensure diversity, each model incorporated from 5 to 25 molecular descriptors randomly selected from the 250 most relevant ones.

Base models were trained using the non-linear Least Squares Support Vector Machines (LSSVM) classification algorithm [37]. To develop LSSVM models, each feature was scaled to the interval [0–1] and the Radial Basis Function (RBF) kernel was used. The optimization of the RBF kernel parameter (σ^2^) and the regularization parameter (γ) was performed by the minimization of the misclassification rate of the 10-fold cross-validated training dataset.

To ensure that each base model was acceptable for further ensemble modeling, we defined few acceptability rules for them. First, a model had to be able of predicting the training data with accuracy not lower than 65%. Also, it had to correctly predict the test data within the model’s applicability domain (AD) with a minimum accuracy of 65%. Finally, a minimum classification accuracy of 65% in five-fold cross-validation experiments has to be achieved.

The AD of the base models is defined according to the molecular descriptors range method [38]. In this case, each feature included in the model is used to build a hyper-rectangle defined by the maximum and minimum value of the features. A sample is considered to be inside the model’s AD if it is included in the defined hyper-rectangle.

### Ensemble modeling

Base models were aggregated into ensembles following two different aggregation strategies: Major vote (MV) and Scores Vote (SV). For MV aggregation, given a set of base models, data is predicted by each model. A sample is assigned to the class (actives *vs* inactives) receiving the higher number of votes [39]. The other aggregation strategy is based on the combination of the classifiers output scores. [39]. For this aggregation strategy the scores produced by the base models are first averaged. Then, if the aggregated score for a compound is greater than zero it is assigned to the actives group and if it is lower than 0 the sample is assigned to the inactive class.

For all strategies, the AD of the base models is considered during the aggregation stage. The AD of the ensemble is defined as the union of the ADs of the base models forming the ensemble. In other words, for a sample to be predicted, only the outputs of the models in the ensemble having it within their AD are aggregated. Thus, for a specific ensemble different models can be aggregated to make decisions on different compounds. Hence, the trained ensembles can be regarded as dynamic.

One of the factors influencing the performance of ensemble models is the diversity of the base models being aggregated [39]. To ensure diversity in the pool of models to be aggregated, a previously developed strategy for base models selection based on Genetic Algorithms (GA) was employed [14,23].

For the GA-guided search of ensembles, each individual represents an ensemble and they are encoded as binary vectors of length 1001 where the “on” bits encode the set of base models considered for the ensemble while “off” bits represent models excluded from the ensemble. The initial population was set to 100 randomly generated individuals and the population evolved for 500 generations. The crossover and mutation rates were set to 0.7 and 0.3, respectively, while the best two individuals survived to the next generation. The selection operator was set to a tournament of size 2. For the crossover operator, the offspring chromosomes were randomly selected, position by position, from the two selected parents. The mutation operator changed a randomly selected “on” bit to “off” and one randomly selected “off” bit to “on” in the individual. For each endpoint, the GA was run five times using different seeded initial populations.

Four fitness functions were explored for the GA-guided selection of the base models forming the ensembles. Two of these fitness functions are the result of maximizing the geometric mean of the BCR metric (see below) across the training and selection sets (BCR_TS_) when the MV and SV methods are employed for models’ aggregation respectively. The other two fitness functions are obtained when Akaike Index (AIC) [40] is minimized for the aforementioned aggregation strategies. For AIC computation *1 –(BCR*_*Train*_*∙BCR*_*Selection*_*)*^*1/2*^ was employed as the ensemble error estimator. The ensemble with the highest value of the geometric mean of the BCR metric across training and selection sets is selected as the optimal one.

### Transformation of scores into desirability values

Ensemble models are employed to predict the classification scores of new compounds. The score of a sample is calculated as the arithmetic mean of the scores predicted by the models having it inside their ADs. These scores will be further transformed into desirability values associated to the ensemble.

For this transformation the aggregated scores for the training, selection and external data sets are computed. These scores are then transformed according to the equation:

$$D_{i}={\left[\frac{{\hat{S}}_{i}-min(S)}{\max\left(S\right)-min(S)}\right]}^{s}$$ (1)

where *Ŝ*_*i*_ is the aggregated score for sample *i*, and *min(S)* and *max(S)* are the minimum and maximum scores for the aggregated scores across training, selection and external data sets. The value of the exponent *s* is determined from the condition that a predicted score equals to 0 corresponds to a value of desirability equals to 0.5, that is, *s* must fulfill the condition:

$$0.5={\left[\frac{-min(S)}{\max\left(S\right)-min(S)}\right]}^{s}$$ (2)

With the aggregated scores for the training, test and external subsets transformed into desirability values and the value of *s* determined, the desirability of any new sample can be computed as:

$$D_{i}=\left\{ \begin{array}{cc} 0 & if\;{\hat{S}}_{i}<min(S)\\ {\left[\frac{{\hat{S}}_{i}-min(S)}{\max\left(S\right)-min(S)}\right]}^{s} & if\;{\min\left(S\right)<\hat{S}}_{i}<max(S)\\ 1 & {\hat{S}}_{i}>max(S) \end{array}\right.$$ (3)

where, *Ŝ*_*i*_ is the aggregated score of the new compound.

### Aggregation of the mapped desirability values into the final multi-criteria decision-making VS tool

With the above transformations, the aggregated score of any chemical can be transformed into desirability values which serve to rank a set of compounds according to the chances of having the desired bioactivity. However, these predictions are only valid for one endpoint. Since the goal of our approach is to use desirability functions for multi-criteria VS, these individual desirability values have to be combined into one predictor comprising information from all endpoints. The combination of the desirability values of individual endpoints is defined by:

Dimult=[∏j=1n(Di,j)wj]1∑j=1nwj (4)

Where *D*_*i*_^*mult*^ is the final multi-criteria desirability for sample *i*, *n* is the number of endpoints considered in the multi-criteria VS experiment, *D*_*i*,*j*_ is the desirability of sample *i* for endpoint *j* and *w*_*j*_ is the weight of endpoint *j* in the whole multi-criteria VS model. According to our previous results [14], all endpoints were given a weight equals to 1.

### Evaluation of the model quality

The quality of the developed models was evaluated by measuring their Accuracy (Acc), Sensitivity (Se), Specificity (Sp) and Balanced Classification Rate (BCR). These metrics are defined as:

$$Acc=\frac{Number\;of\;correctly\;classified\;compounds}{Total\;number\;of\;compounds}$$ (5)

$$Se=\frac{Number\;of\;correctly\;classified\;active\;compounds}{Total\;number\;active\;compounds}$$ (6)

$$Sp=\frac{Number\;of\;correctly\;classified\;inactive\;compounds}{Total\;number\;inactive\;compounds}$$ (7)

$$BCR=\frac{Se+Sp}{2}*(1-|Se-Sp|)$$ (8)

### Evaluation of the VS performance of the obtained models

To evaluate the performance of the developed models in a VS scenario the following metrics were computed: Area Under the Accumulation Curve (AUAC); area under the Receiver Operating Characteristic Curve (ROC); Enrichment Factor (EF) and Boltzmann-Enhanced Discrimination of ROC (BEDROC) [41,42] according to the definitions proposed by Truchon *et al*.[41]. In brief, let us consider a ranking of *N* compounds where *n* are active and each active has a ranking position *r*_*i*_ and a relative ranking *x*_*i*_ *= r*_*i*_*/N* in the whole ranked list. Given these definitions, the area under the accumulation curve AUAC is computed as:

$$AUAC=1-\frac{1}{n}\sum_{i=1}^{n}x_{i}$$ (9)

It has also been shown that AUAC and ROC are related by the equation:

$$ROC=\frac{AUAC}{R_{i}}-\frac{R_{a}}{2R_{i}},\;where\;R_{i}=\frac{N-n}{N}\;and\;R_{a}=\frac{n}{N}$$ (10)

For a perfect ranking ROC = 1 while ROC = 0.5 corresponds to a uniform distribution of the actives in the ranked list.

The EF metric is defined as the number of active compounds found in a fraction 0 < χ ≤ 1 of the ordered list relative to what is expected from a uniform ranking for that same fraction:

$$EF=\frac{\sum_{i=1}^{n}\delta_{i}}{\chi n},\;where\;\delta_{i}=\left\{ \begin{array}{c} 1\;r_{i}\leq \chi N\\ 0\;r_{i}>\chi N \end{array}\right.$$ (11)

The maximum value that EF can take is 1 ∕ χ if χ ≥ n ∕ N and N ∕ n if χ < n ∕ N. The minimum value for EF is 0.

AUAC and ROC are not appropriate to address the early recognition problem. On the other hand, although correctly ranking VS methods, EF has the disadvantage that it equally weights all the active compounds within the considered cutoff.

Another metric used to evaluate the performance of VS metrics is the Robust Initial Enhancement (RIE) [43]. This metric is defined as:

$$RIE=\frac{\frac{1}{n}\sum_{i=1}^{n}e^{-\alpha x_{i}}}{\frac{1}{N}(\frac{1-e^{-\alpha }}{e^{\alpha /N}-1})}$$ (12)

The effect of the α parameter is that the active compounds ranked at the beginning of the ordered list get higher weights than those at the tail and will then make a higher contribution to the RIE metric. Despite addressing the early recognition problem, the RIE metric has the disadvantages of being highly dependent on *n* and *N* and of not been bounded. To overcome these difficulties of the above presented metrics the Boltzmann-Enhanced Discrimination of ROC (BEDROC) metric was proposed [41]. This metric is defined as:

$$BEDROC=\frac{RIE-{RIE}_{min}}{{RIE}_{max}-{RIE}_{min}}$$ (13)

$${RIE}_{min}=\frac{1-e^{\alpha R_{a}}}{R_{a}(1-e^{\alpha })}\;and\;{RIE}_{max}=\frac{1-e^{-\alpha R_{a}}}{R_{a}(1-{e-}^{\alpha })}$$

are obtained from the definition of RIE (see Eq 12) when all the actives are at the beginning and at the tail of the ranked list respectively.

The BEDROC metric, as well as AUAC and ROC, is bounded by 0 and 1. The parameter α is derived from the equation:

$$\theta (1-e^{-\alpha })-1+e^{-\alpha z}=0$$ (14)

To compute α from this equation it has to be considered that *z* represents the percent of the ranked list at which enrichment is important and *θ* is the expected contribution of the enrichment at this *z*% fraction to the overall enrichment. For example, if enrichment is important at the first 1% (*z*) of the ranked list and this fraction is expected to contribute with 80% (θ) of the overall enrichment, then α takes a value equals to 160.9.

## Results and discussion

### Data sets and molecular descriptors

**Fig 1:**
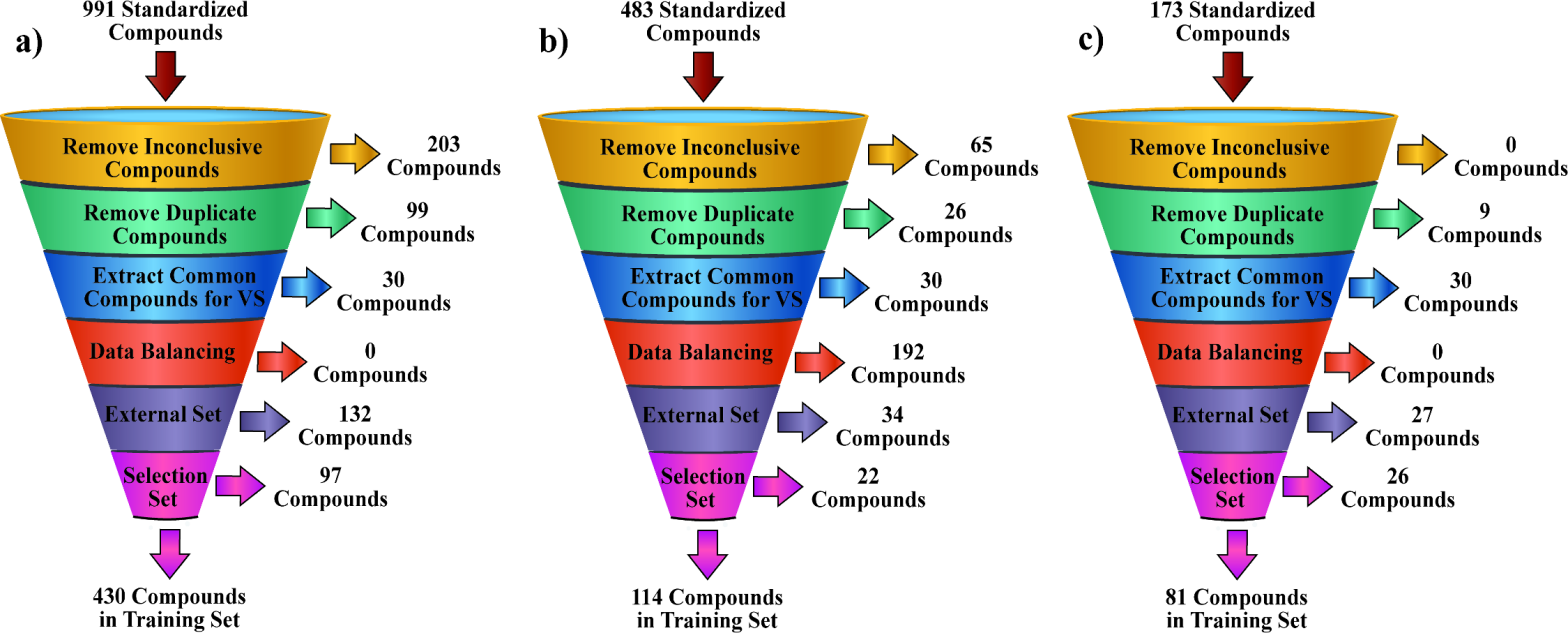
Summary of the data sets curation workflow. Results are summarized for the: a) AGS, b) NCI-N87 and c) SNU-1 data sets.

Data sets were collected and curated following the procedure described in the Computational Methods section. The curation procedure is summarized in Fig 1. The raw data sets contained 931, 418 and 173 compounds for the AGS, NCI-N87 and SNU-1 cell lines respectively. The removal of the samples which could not be unambiguously assigned to one class resulted in 203 compounds being removed from the AGS data set and 65 from the NCI-N87 one. No compound was removed from the SNU-1 data set.

Afterward, duplicate samples were identified and removed from the data set. This resulted in 99, 26 and 9 compounds being removed from the AGS, NCI-N87 and SNU-1 data sets respectively. Next, the set of compounds tested in all the three tumor cell lines was extracted and from them 15 from each class were reserved for the VS validation experiments.

It is a well-known fact that large unbalance among classes can negatively affect the performance of machine learning algorithms. After applying the above described filters, the AGS and SNU-1 data sets contained 40% of actives each, which cannot be considered a high level of unbalance. However, the NCI-N87 data set contained 85 inactive and 277 active compounds yielding 23% of inactives in this set. To avoid the negative effects of this unbalance during the modeling process, a representative subset of 85 active compounds was selected for the NCI-N87 data set by employing the Maximal-Minimal Dissimilarity Selection algorithm implemented on the ChemAxon’s JKlustor software [33].

Next, 20% of the compounds of each class for the three data sets were selected for the external validation set. The remaining chemicals were split into the training and selection sets employing the Sphere Exclusion algorithms. This procedure yielded training sets of size 430, 114 and 81; selection sets of size 97, 22 and 26; and external validation sets of size 132, 34 and 27 for the AGS, NCI-N87 and SNU-1 data sets respectively. In the same way as the aforementioned unbalance between classes, data representativeness can also influence the performance of machine learning algorithms. In consequence, it should be expected a better classification performance for the AGS data set. The SD files of the final training, selection and external sets for the three data sets are provided as Supporting Information in the S1 File.

Molecular descriptors were computed and filtered for the training data set and the subset of 250 most relevant descriptors was calculated for the selection and external subsets for each of the AGS, NCI-N87 and SNU-1 data sets (S2 File in Supporting Information).

### Base models

The mean performances of the base models as well as of the best performing base model per endpoint are summarized in Table 1. The best base model was selected as the one with the maximum value of geometric mean of the BCR metric in the training and selection data sets.

**Table 1:** Performance of the base models.

	Classification Performance				BCR		
	Train ^(^^a^^,^^e^^)^	Selection ^(^^b^^,^^e^^)^	External ^(^^c^^,^^e^^)^	Cross-validation Accuracy ^(d)^	Train ^(a)^	Selection ^(b)^	External ^(c)^
AGS							
Mean	84 (67/96)	77 (60/89)	73 (58/84)	74.06	58.44	53.02	52.29
Best Model	97 (96/98)	85 (83/87)	72 (76/69)	71.86	94.67	81.4	67.7
NCI-N87							
Mean	89 (88/89)	74 (82/67)	56 (57/55)	73.93	82.57	58.88	46.97
Best Model	100 (100/100)	82 (82/82)	47 (53/41)	78.07	100.00	81.82	41.52
SNU-1							
Mean	82 (62/96)	68 (40/86)	52 (24/72)	70.66	54.43	33.97	24.56
Best Model	98 (97/100)	80 (78/81)	56 (64/50)	72.84	95.50	76.75	49.07

^(a)^ Training data set.

^(b)^ Selection data set.

^(c)^ External data set.

^(d)^ Cross-validation accuracy

^(e)^ Results are presented as Accuracy (Sensitivity/Specificity)

As can be seen from Table 1 the mean performance of the base models was similar across the three modeled endpoints for the training and selection datasets. However, the mean performance of the base models on the external set was much lower for the NCI-N87 and SNU-1 data sets than for the AGS endpoint. In addition, it can be seen that, in average, in most cases the prediction of the data sets resulted in unbalanced predictions according to the observed mean values of Sensitivity and Specificity. In the same way, particularly for the NCI-N87 and SNU-1 datasets, the best performing model showed very poor generalization ability with either Sensitivity or Specificity being worse than for a random model. These results show that even the best performing base model is only able to generalize for the AGS endpoint. This behavior can be related to the fact that the AGS data set is much more informative that the NCI-N87 and SNU-1 ones.

### Ensemble modeling

For each endpoint, base models were aggregated using the GA-guided optimization previously described. For each combination of aggregation strategy (MV, SV) and objective function (BCR_TS_, AIC) the GA optimization was run constraining the maximum number of allowed base models in each individual of the initial population to 5, 10 and 15. This yields 12 different combinations of the GA-optimization settings for the ensemble modeling of each endpoint. The performance statistics of the best ensemble model as well as the mean performance of the best 12 models per endpoint (one per settings combination) are presented in Table 2. The full performance statistics for the 12 models per endpoint are presented in the Supporting Information S1 Table.

**Table 2:** Summary of the performance of the developed ensemble models.

	Classification Performance			BCR			
	Train^(^^a^^,^^e^^)^	Selection^(^^b^^,^^e^^)^	External^(^^c^^,^^e^^)^	Train^(a)^	Selection^(b)^	External^(c)^	$\sqrt{Train*Sel.}$^(d)^
AGS							
Mean	98 (95/100)	86 (85/87)	78 (78/78)	93.02	83.21	77.58	87.96
Best Model	99 (98/99)	87 (85/88)	77 (77/78)	96.88	84.59	76.77	90.53
NCI-N87							
Mean	99 (99/99)	81 (85/76)	71 (71/71)	97.69	70.73	69.84	82.89
Best Model	100 (100/100)	91 (91/91)	71 (71/71)	100.00	90.91	70.59	95.35
SNU-1							
Mean	98 (97/100)	85 (80/88)	64 (63/65)	94.08	71.67	61.60	81.47
Best Model	99 (97/100)	100 (100/100)	63 (64/63)	95.50	100.00	62.35	97.72

^(a)^ Training data set.

^(b)^ Selection data set.

^(c)^ External data set.

^(d)^ Geometric mean of the BCR metric across training and selection sets

^(e)^ Results are presented as Accuracy (Sensitivity/Specificity)

As can be seen from Table 2, the best performance was obtained for the AGS dataset, followed by the NCI-N87 and SNU-1 data sets. This better performance might be related to the larger data set available for modeling the AGS endpoint. Nevertheless, in all cases both the mean performance and the performance of the best ensemble model showed improved generalization compared to the best performing base model (see Table 1). Despite away from the performance obtained for the AGS data set, the implemented ensemble modeling strategy provided, in contrast to the base models, generalizable models for the NCI-N87 and SNU-1 data sets. More important, the classification statistics of each group in all the three data sets (Sensitivity and Specificity) are balanced, an indication that the developed models will provide the same rate of false positives/negatives in the prediction of new chemicals.

**Fig 2:**
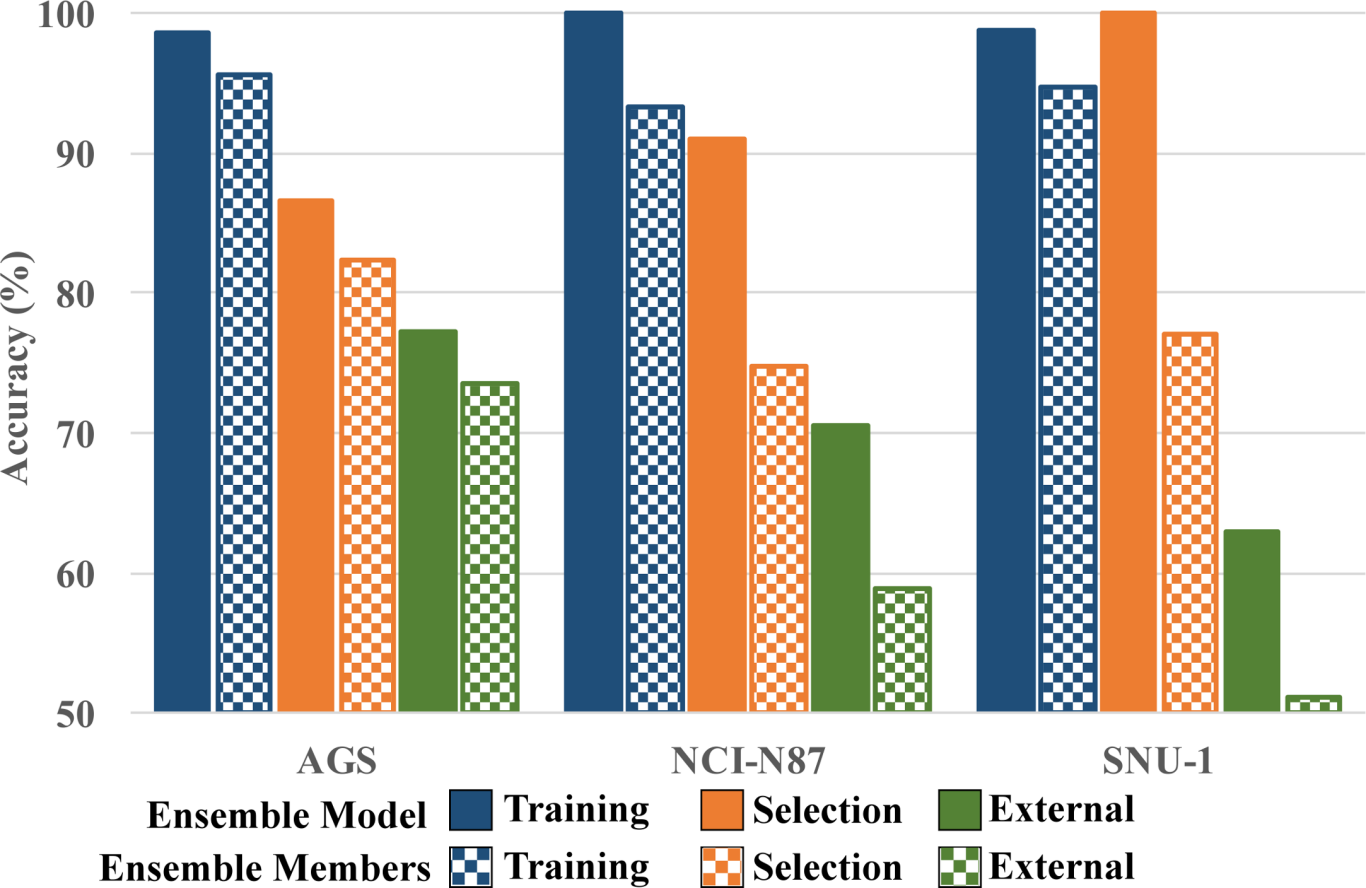
Comparison between the accuracy of the best ensemble for each endpoint and the mean accuracy of the base models they are composed by. The mean accuracy of the base models is shown using squares-pattern bars.

To get more insights into the performance of the developed ensemble models, we compared their classification performance to that of the base models they are composed by. The mean performance statistics for the base models included in each ensemble are provided in the Supporting Information S2 Table. The comparison corresponding to the best ensemble model per endpoint is summarized in Fig 2.

The best performing ensemble for the AGS endpoint was obtained for the GA setup consisting on the aggregation of the base models using the SV method, constraining the number of base models in the initial population to a maximum of five and minimizing AIC. For the NCI-N87 cell line, the setup yielding the best performing ensemble consisted on the aggregation of the base models using SV with a maximum of 10 base models in the initial population and maximizing the BCR_TS_ metric. Finally, for the SNU-1 endpoint the best performing ensemble was obtained with the optimization of the BCR_TS_ metric when the base models were aggregated using the MV strategy and the number of base models in the initial population was constrained to five. The size of the best performing ensemble was 5, 8 and 7 models for the AGS, NCI-N87 and SNU-1 data sets, respectively. This last observation highlights the well-known fact that aggregating a small subset of diverse base models can be an effective strategy for obtaining good performing ensemble models.

The three best performing ensemble models presented in Fig 2outperform the classification accuracy in predicting the training, selection and external data sets relative to the average of the base models they are composed by. If the generalization capabilities (measured as the classification accuracy on the external data set) of these models is analyzed it can be seen that the lower improvement is obtained for the AGS endpoint. In this case the improvement in classification accuracy is 3.76%. Although not very high, this improvement comes with a better balance between Sensitivity and Specificity compared to the ensemble members.

On the other hand, the improvement in the accuracy in predicting the external set is 11.69% and 11.84% for the NCI-N87 and SNU-1 data sets, respectively. This represents a large improvement relative to the base models included in these ensembles which showed no generalization capabilities.

All these evidences show that ensemble modeling is a robust approach for obtaining accurate and predictive QSAR models. This observation is in agreement with similar conclusions that have been derived in previous research [14,23,44,45]. By following this approach it is possible to obtain predictive models for the classification-based modeling of the GC cell lines employed in this research. These ensemble models will serve for establishing the multi-objective approach for the VS of databases of chemical compounds in the search of broad-spectrum anti-GC agents.

### Ensemble-based VS simulations

The main goal of many VS campaigns is the discovery of compounds with the desired bioactivity. It has been reported that good classification models are not necessarily good VS tools [45]. For this reason, it has been proposed that the validation of models in VS conditions should become a required step for the evaluation of computational models intended for VS. In this research, the desirability-based approach previously proposed by us [14] was employed to search the best performing multi-objective VS model.

In our previous research, the best ensemble model for each endpoint was employed to predict the scores of the samples within the ensemble’s AD which are then transformed into desirability scores. Finally, the multi-objective desirability was computed as the geometric mean of the desirability scores across all endpoints. Here, instead of limiting to the aggregation of the best ensemble model per endpoint, we explored all the possible combinations of the 12 final ensembles trained for each endpoint. As a consequence, the VS performances of the 1728 possible combinations of ensemble models were explored.

As discussed above, the data curation process included the reservation of 15 representative active compounds for VS simulations. These were selected from the whole pool of chemicals classified as actives for all the three endpoints, i.e. compounds with anti-tumoral activity against the three GC cell lines included in this study. Given that the number of confirmed inactives available for VS simulations is very limited, decoy molecules for these 15 active molecules were generated using the DUD-E server [34]. This resulted in the selection of 950 decoy molecules for VS validation experiments. The VSVS consisting on 980 compounds was hence composed by the 15 representative actives, the selected decoy molecules and the previously reserved 15 representative compounds simultaneously inactive against the AGS, NCI-N87 and SNU-1 cell lines. The SD file of the VSVS is provided in the Supporting information S3 File.

The scores of each compound in the VSVS for the 12 selected ensemble models per endpoint were computed and transformed into desirabilities. For each possible fusion of the ensembles-derived desirabilities the BEDROC, EF and AUAC enrichment metrics were computed. This search provided 1728 possible multi-objective VS protocols from which we can select the most suitable one according to the VS problem being addressed.

A VS campaign can be oriented toward different objectives. First of all, depending on the number of screened compounds and the available experimental resources, a researcher would need to select different top-fractions of screened data. Once the size of the subset of top-ranked molecules to be selected has been defined, different scenarios are possible. One of these scenarios is that the proposed VS protocol is the final VS tool. In that case it is important that the active compounds in that fraction of screened data appear as close as possible to the first place in the ranked list. According to that, the BEDROC metric (suitable for detecting early enrichment) is required to be maximized.

Other possible scenario is that the VS tool is employed to maximize the amount of active compounds retrieved at a given top-fraction of screened data. That would be the case when a VS tool is employed as an intermediate filter for further processing. In this case it is important that the selected VS protocol maximizes the EF metric at a given fraction of screened data. The flexibility of the methodology herein proposed makes it suitable for any of these scenarios.

Given the large amount of potential analyses that can be derived from these possibilities, we focus our analyses on the enrichment optimization for a selection size equals to 1% of screened data. In this case scenario 18 out of the 1728 assayed combinations of endpoints’ ensembles share the same maximum value of EF = 19.6. This means that any of these multi-objective VS protocols yield 19.6 times more actives in the top 1% of screened data than what is expected from a uniform distribution of the actives in the list. The summary of the EF, BEDROC and AUAC metrics for these VS protocols as well as the model of each endpoint they are formed by are presented in Table 3. Also, the coverage of the VSVS by the VS protocols ADs is included in Table 3.

**Table 3:** VS performance of the multi-target protocols.

VS Protocol	Model^(a)^			EF 1%^(b)^	BEDROC (α = 160.9) ^(c)^	AUAC^(d)^	Cov. Dom. (%)^(e)^
	AGS	NCI-N87	SNU-1				
1	MV-5-BCR_TS_	MV-5- BCR_TS_	MV-15- BCR_TS_	19.60	0.35	0.54	100
2	MV-5- BCR_TS_	MV-5- BCR_TS_	MV-15-AIC	19.60	0.35	0.58	100
3	MV-5- BCR_TS_	MV-5- BCR_TS_	SV-10- BCR_TS_	19.60	0.29	0.60	100
4	MV-10- BCR_TS_	SV-5- BCR_TS_	MV-15- BCR_TS_	19.60	0.26	0.62	100
5	SV-5- BCR_TS_	MV-5- BCR_TS_	MV-15- BCR_TS_	19.60	0.25	0.54	100
6	SV-5- BCR_TS_	MV-5- BCR_TS_	MV-15-AIC	19.60	0.31	0.59	100
7	SV-15-AIC	MV-5- BCR_TS_	MV-5- BCR_TS_	19.60	0.27	0.59	100
8	SV-15-AIC	MV-5- BCR_TS_	MV-15- BCR_TS_	19.60	0.41	0.57	100
9	SV-15-AIC	MV-5- BCR_TS_	MV-15-AIC	19.60	0.32	0.61	100
10	SV-15-AIC	MV-5- BCR_TS_	SV-10- BCR_TS_	19.60	0.43	0.64	100
11	SV-15-AIC	MV-5- BCR_TS_	SV-15- BCR_TS_	19.60	0.38	0.57	100
12	SV-15-AIC	MV-10- BCR_TS_	MV-15- BCR_TS_	19.60	0.23	0.55	100
13	SV-15-AIC	MV-15- BCR_TS_	MV-15- BCR_TS_	19.60	0.35	0.57	100
14	SV-15-AIC	MV-15- BCR_TS_	MV-15-AIC	19.60	0.26	0.62	100
15	SV-15-AIC	MV-15- BCR_TS_	SV-10- BCR_TS_	19.60	0.35	0.64	100
16	SV-15-AIC	SV-5- BCR_TS_	MV-15- BCR_TS_	19.60	0.33	0.56	100
17	SV-15-AIC	SV-5- BCR_TS_	SV-10- BCR_TS_	19.60	0.35	0.65	100
18	SV-15-AIC	SV-10- BCR_TS_	MV-15- BCR_TS_	19.60	0.25	0.59	100

^(a)^ Model of each endpoint aggregated for the VS protocol. The code of each model is based upon the combination of Aggregation Method (MV, SV), Number of Allowed Base Models in the Initial Population (5, 10, 15) and Minimized Metric (AIC, Classification Error)

^(b)^ EF at a selection size equals to 1% of screened data

^(c)^ BEDROC for α = 160.9

^(d)^ Area Under the Accumulative Curve

^(e)^ Percent of coverage of the VSVS by the multi-objective VS protocol AD

Some of the ensemble models for the three endpoints repeatedly appear among the best performing VS protocols. From Table 3 it can be seen that for the AGS dataset, the ensemble obtained when SV is used for aggregation, 15 maximum models are allowed in the initial population and AIC is minimized (SV-15-AIC) appears in 12 out of the 18 top performing VS protocols. In the same way, the ensemble obtained when the MV is used as aggregation strategy, having 5 maximum base models in the initial population and optimizing the BCR_TS_ metric (MV-5- BCR_TS_), appears in 10 of the best performing VS protocols. Finally, for the SNU-1 endpoint, the most frequent ensemble in the best performing VS protocols is the one formed when MV is employed for aggregation, the maximum number of base models in the initial population is set to 15, and BCR_TS_ is maximized (MV-15- BCR_TS_).

**Fig 3:**
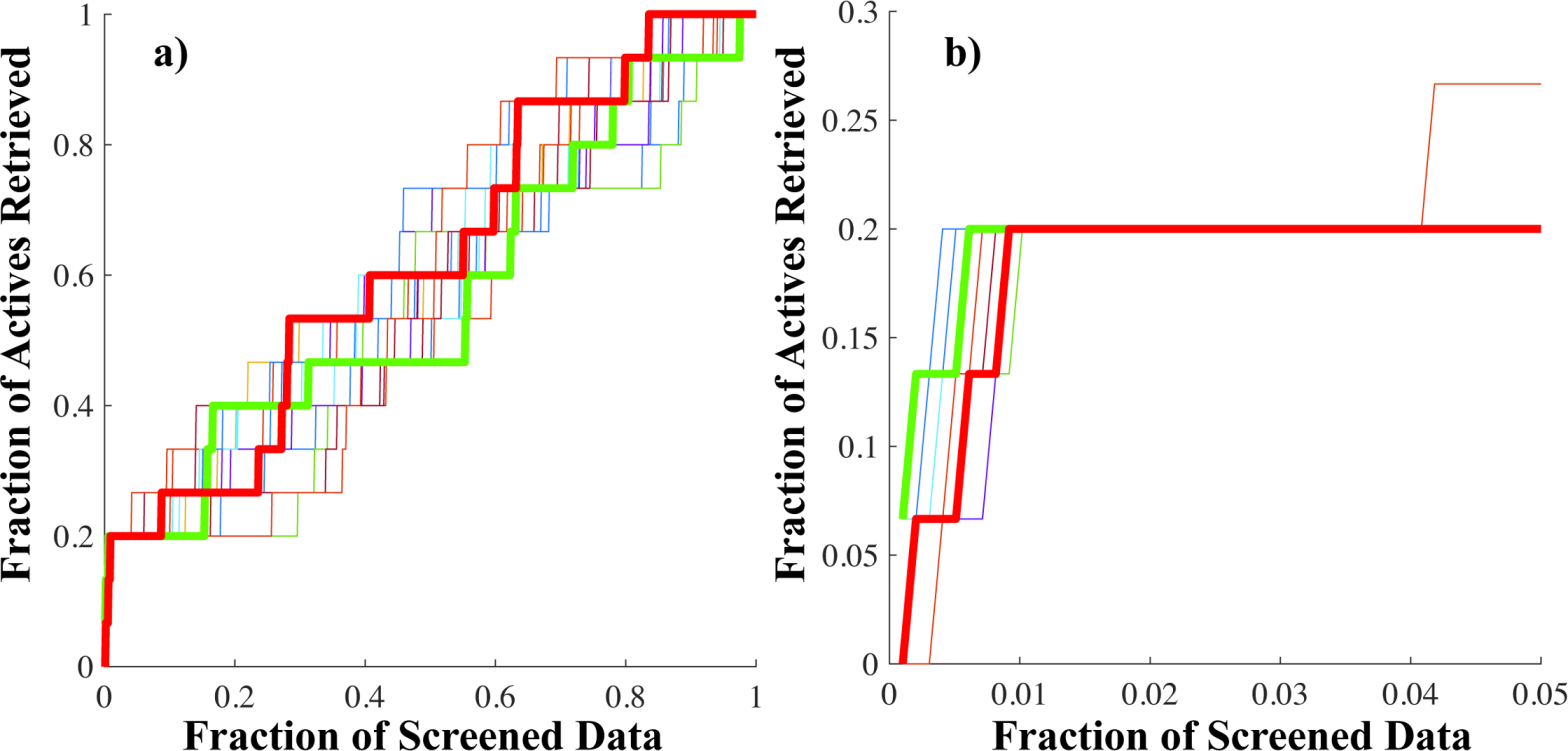
Accumulative curves for the 18 top-performing VS protocols. Results are presented for a) Whole screening. b) Top 5% of screened data.

Apparently, all these VS protocols are equivalent; however, this false impression comes from the inability of the EF metric to measure the early enrichment of a VS method. If the BEDROC metric when 80% of the screening importance comes from the first 1% of screened data (α = 160.9) is analyzed, one of these 18 VS protocols stands over the rest. VS protocol 10 outperforms the early enrichment ability of the rest of the top performing models. This behavior can be better observed in Fig 3 where the accumulative curves for these 18 top-performing VS protocols are reported.

In Fig 3 the accumulative curves corresponding to VS protocol 10 (best early enrichment) and VS protocol 12 (worst early enrichment) are represented using thick lines and colored green and red, respectively. All these 18 VS protocols are able to retrieve 3 actives compounds in the first 1% of screened data (10 molecules). Apparently, there are no differences between the accumulative curves for these VS protocols at their early parts (Fig 3 (A)). However, a closer look at the first 5% of the ranked lists (Fig 3 (B)) reveals that VS protocol 10 retrieves these active compounds in the first 3 ranking positions. In consequence, it achieves the highest value of BEDROC among the analyzed VS protocols. In contrast, VS protocol 12 identifies the 3 active compounds in the first 1% of screened data at positions 3, 6 and 10. All these evidences support the selection of VS protocol 10 for the discovery of chemicals with simultaneous anti-tumoral activity against the GC cell lines AGS, NCI-N87 and SNU-1 through VS. Further analyses were performed for VS protocol 10 in order to get more insights into its performance.

To get further insights into the performance of VS protocol 10, we analyzed the positions that the 15 inactives reserved for VS validations occupy in the ranked list that it produces. In this case no confirmed inactive compound appears in the first 1% of screened data. The first confirmed inactive molecule appears in position 13 (close to the top 1% of screened data).The next confirmed inactive compound is ranked in position 182 which is beyond the 18% of screened data for the VSVS. The rest of the confirmed inactives occupy ranking positions beyond the 40% of screened data. In summary: 1) no confirmed inactive compound is included in the first 1% of the ranked list, and 2) only 2 confirmed inactive compounds are ranked among the first 20% of screened data (a selection fraction large enough for the VS of small databases of chemicals).

Regarding the coverage of the VSVS by the AD of VS protocol 10, it has all compounds in the VSVS within its AD. This is an indication that the AD of VS protocol 10 is not a restrictive filter leading to artificial enrichment values. As previously suggested [14,23], this behavior is one of the advantages of employing ensemble modeling for multi-objective VS protocols over the use of individual models as predictors for each property. For a chemical to be within the AD of one of the explored VS protocols, it has to simultaneously be inside the AD of each endpoint’s ensemble model. In the case of VS protocol 10, this condition is met for the whole VSVS.

It is noteworthy that none of the base models included in any of the endpoint’s ensembles used for VS protocol 10 has 100% coverage of the VSVS by its AD. The fact that the three ensembles used for VS protocol 10 have the 100% of the VSVS inside their ADs is due to the definition of the ensembles’ ADs as the union of the base models’ ADs. That is, to be within the ensemble’s AD it is only necessary that the compound is inside the AD of at least one of the base models composing the ensemble.

One of the objectives of our research is to discover molecules simultaneously effective against the GC cell lines AGS, NCI-N87 and SNU-1. In future research, we will perform the VS of databases of chemical compounds and the experimental evaluation of the most promising candidates. Although no experimental evaluation has been performed at this stage, we completed the VS of the DrugBank database [46] employing VS protocol 10. Some of the results that we obtained can be considered promising and they can support the utility of our VS pipeline.

DrugBank was prepared for VS following the same procedures as above. Results are only commented for the curated database and for compounds inside the AD of VS protocol 10. When the top 1% of the ranked database (83 molecules) is analyzed it can be seen that the sample ranked in the first position (DB00570) shares high structural similarity with compounds present in our datasets and that it has been experimentally corroborated active against the three GC cell lines under investigation. The entry ranked in second position was already present in our data sets, however, the chemical ranked in the third position (DB11752) is not included in our data sets and in recent ChEMBL versions it is annotated as active against the GC cell lines AGS, NCI-N87 and SNU-1.

We also evaluated the enrichment in the top 1% of the ranked list with reported anti-neoplastic agents. For this, we searched for compounds labeled as “ANTINEOPLASTIC AGENTS” according to the WHO drug classification system in the ranked DrugBank list. This analysis conducted to 125 compounds reported as anti-neoplastic in the whole database, from them, 12 were ranked in the top 1% of the list. These amounts represent 1,51% and 14,45% of reported anti-neoplastic agents in the whole database and the top 1% of the ranked list, respectively. Overall, this means that VS protocol 10 enriches the first 1% of the screened DrugBank with 9.55 times more known anti-neoplastic agents than the whole database. Considering that DrugBank contains only drugs and drug candidates in late development stages this enrichment can be considered relevant. This result encourages us to experimentally evaluate the top ranked compounds from DrugBank for their in vitro efficacy in future research.

Based on the results here discussed, VS protocol 10 can be selected as the best performing VS tool for the discovery of broad-spectrum anti-GC chemicals. The parameters required for training the LSSVM base models included in VS protocol 10 are provided in the Supporting information S3 Table.

Two final considerations have to be taken into account. Firstly, as for experimental trial and error methods, when employing VS as filter in drug discovery there are the risks of: 1) selecting compounds which are predicted as active but display no bioactivity in the lab (False Positives) and 2) not selecting for experiments compounds having the desired bioactivity (False Negatives). These risks are related to the methods’ inherent limitations such as inconsistencies on data due to the inaccurate reports of bioactivity on databases (e.g. ChEMBL), overfitting, limited applicability domain, insufficient validation of the models prior to their use and the use of incorrect modeling practices. To reduce the incidence of these factors, all modeling steps in this research were carefully performed following state of the art methods and guidelines. We also performed VS validations and the VS of the DrugBank database, obtaining results that support the utility of VS protocol 10 for the discovery of broad-spectrum anti-GC molecules. The final success of the proposed VS workflow can only be established through the discovery of effective in vitro broad-spectrum anti-GC agents. These experimental corroborations will be the objective of our forthcoming research efforts.

Secondly, it has to be considered that the proposed approach is based on the phenotypic response of GC to chemical compounds. That is, we focused on modeling the final anticancer activity without taking into account molecules’ mode of action. Once multi-target GC inhibitors are identified, further studies will be necessary to decipher their mode of action. This will allow to establish whether the mode of action of the hit molecules is the same on all GC cell lines or not. Such information will be essential for optimizing the activity and pharmacological properties of the discovered lead molecules.

## Conclusions and further work

Here, we have presented the application of a workflow relying on our recently reported methodology based on ensemble modeling and desirability [14] for exploring VS models to identify broad-spectrum anti-GC chemicals. Bioactivity data for the GC cell lines AGS, NCI-N87 and SNU-1 were collected from the ChEMBL database and subjected to a thorough curation process. Ensemble modeling led to accurate and generalizable QSAR models. These ensemble models were then used for exploring possible multi-objective VS protocols. The best VS protocol was able to achieve high values of enrichment in VS simulations, proving that our approach could be an effective tool for the rational discovery of broad-spectrum anti-GC chemicals. The results obtained in the VS of the DrugBank database also supported this conclusion. This VS protocol will be applied to the VS identification of potential broad-spectrum anti-GC chemicals from databases of commercially available chemical compounds. These predictions will be evaluated in the wet-lab in further research stages.

## Supporting information

S1 Table Performance statistics for the 12 ensemble models selected per endpoint. (DOCX)

S2 Table Mean performance statistics for the base models included in each ensemble. (DOCX)

S3 Table Parameters for the LS-SVM base models included in VS protocol 10. (DOCX)

S1 File SD files of the final training, selection and external sets for the three data sets. (ZIP)

S2 File List of the final 250 ISIDA fragments computed for each of the AGS, NCI-N87 and SNU-1 data sets. (ZIP)

S3 File SD file of the virtual screening validation set. (ZIP)
