# Supplementary material for: A desirability-based multi objective approach for the virtual screening discovery of broad-spectrum anti-gastric cancer agents
Source: PLoS One. 2018 Feb 8;13(2):e0192176. doi: 10.1371/journal.pone.0192176 (PMC5805264; doi:10.1371/journal.pone.0192176)
Supplement: S1 Table — (DOCX) [file pone.0192176.s001.docx]

# **S1 Table. Performance statistics for the 12 ensemble models selected per endpoint.**

|  | |  | |  | |  | | Accuracy | | | | | | Sensitivity | | | | | | Specificity | | | | | | BCR | | | | | | | |
| --- | --- | --- | --- | --- | --- | --- | --- | --- | --- | --- | --- | --- | --- | --- | --- | --- | --- | --- | --- | --- | --- | --- | --- | --- | --- | --- | --- | --- | --- | --- | --- | --- | --- |
| Aggregation^(e)^ | | Max. Models Initial Population | | Objective Function | | Size | | Train^(a)^ | | Sel. ^(b)^ | | Ext. ^(c)^ | | Train^(a)^ | | Sel. ^(b)^ | | Ext. ^(c)^ | | Train^(a)^ | | Sel. ^(b)^ | | Ext. ^(c)^ | | Train^(a)^ | | Sel. ^(b)^ | | Ext. ^(c)^ | | $\sqrt{Train*Sel.}$^(d)^ | |
| **AGS** | | | | | | | | | | | | | | | | | | | | | | | | | | | | | | | | |  |
| MV | 5 | | Error | | 5 | | 0.9791 | | 0.8763 | | 0.7803 | | 0.9529 | | 0.8780 | | 0.7885 | | 0.9962 | | 0.8750 | | 0.7750 | | 0.9324 | | 0.8739 | | 0.7712 | | 0.9027 | |  |
| MV | 10 | | Error | | 7 | | 0.9721 | | 0.8660 | | 0.7879 | | 0.9412 | | 0.8537 | | 0.7885 | | 0.9923 | | 0.8750 | | 0.7875 | | 0.9173 | | 0.8459 | | 0.7872 | | 0.8809 | |  |
| MV | 15 | | Error | | 5 | | 0.9791 | | 0.9072 | | 0.7803 | | 0.9588 | | 0.8780 | | 0.7885 | | 0.9923 | | 0.9286 | | 0.7750 | | 0.9429 | | 0.8577 | | 0.7712 | | 0.8993 | |  |
| MV | 5 | | AIC | | 5 | | 0.9698 | | 0.8763 | | 0.7955 | | 0.9294 | | 0.8537 | | 0.7885 | | 0.9962 | | 0.8929 | | 0.8000 | | 0.8985 | | 0.8390 | | 0.7851 | | 0.8683 | |  |
| MV | 10 | | AIC | | 5 | | 0.9791 | | 0.8969 | | 0.7803 | | 0.9529 | | 0.8780 | | 0.7692 | | 0.9962 | | 0.9107 | | 0.7875 | | 0.9324 | | 0.8652 | | 0.7641 | | 0.8982 | |  |
| MV | 15 | | AIC | | 3 | | 0.9837 | | 0.8454 | | 0.7727 | | 0.9588 | | 0.8293 | | 0.7692 | | 1.0000 | | 0.8571 | | 0.7750 | | 0.9391 | | 0.8197 | | 0.7677 | | 0.8774 | |  |
| SV | 5 | | Error | | 4 | | 0.9767 | | 0.8660 | | 0.7727 | | 0.9529 | | 0.8293 | | 0.7692 | | 0.9923 | | 0.8929 | | 0.7750 | | 0.9343 | | 0.8063 | | 0.7677 | | 0.8680 | |  |
| SV | 10 | | Error | | 3 | | 0.9884 | | 0.8454 | | 0.7727 | | 0.9765 | | 0.8293 | | 0.7692 | | 0.9962 | | 0.8571 | | 0.7750 | | 0.9669 | | 0.8197 | | 0.7677 | | 0.8903 | |  |
| SV | 15 | | Error | | 3 | | 0.9884 | | 0.8351 | | 0.7955 | | 0.9706 | | 0.8537 | | 0.7885 | | 1.0000 | | 0.8214 | | 0.8000 | | 0.9563 | | 0.8105 | | 0.7851 | | 0.8804 | |  |
| SV | 5 | | AIC | | 5 | | 0.9860 | | 0.8660 | | 0.7727 | | 0.9765 | | 0.8537 | | 0.7692 | | 0.9923 | | 0.8750 | | 0.7750 | | 0.9688 | | 0.8459 | | 0.7677 | | 0.9053 | |  |
| SV | 10 | | AIC | | 2 | | 0.9512 | | 0.8144 | | 0.7879 | | 0.8882 | | 0.8049 | | 0.7885 | | 0.9923 | | 0.8214 | | 0.7875 | | 0.8424 | | 0.7997 | | 0.7872 | | 0.8208 | |  |
| SV | 15 | | AIC | | 5 | | 0.9814 | | 0.8454 | | 0.7879 | | 0.9529 | | 0.8780 | | 0.7885 | | 1.0000 | | 0.8214 | | 0.7875 | | 0.9305 | | 0.8016 | | 0.7872 | | 0.8637 | |  |
| Mean |  | |  | | 4.33 | | 0.9779 | | 0.8617 | | 0.7822 | | 0.9510 | | 0.8516 | | 0.7804 | | 0.9955 | | 0.8690 | | 0.7833 | | 0.9302 | | 0.8321 | | 0.7758 | | 0.8796 | |  |
| **NCI-N87** | | | | | | | | | | | | | | | | | | | | | | | | | | | | | | | | |  |
| MV | 5 | | Error | | 5 | | 0.9912 | | 0.8182 | | 0.6471 | | 1.0000 | | 0.9091 | | 0.6471 | | 0.9825 | | 0.7273 | | 0.6471 | | 0.9738 | | 0.6694 | | 0.6471 | | 0.8074 | |  |
| MV | 10 | | Error | | 19 | | 0.9912 | | 0.8182 | | 0.7059 | | 0.9825 | | 0.8182 | | 0.7059 | | 1.0000 | | 0.8182 | | 0.7059 | | 0.9738 | | 0.8182 | | 0.7059 | | 0.8926 | |  |
| MV | 15 | | Error | | 7 | | 0.9912 | | 0.8571 | | 0.7059 | | 1.0000 | | 0.8182 | | 0.7059 | | 0.9825 | | 0.8182 | | 0.7059 | | 0.9738 | | 0.8182 | | 0.7059 | | 0.8926 | |  |
| MV | 5 | | AIC | | 3 | | 0.9825 | | 0.7727 | | 0.7059 | | 0.9825 | | 0.8182 | | 0.7059 | | 0.9825 | | 0.7273 | | 0.7059 | | 0.9825 | | 0.7025 | | 0.7059 | | 0.8308 | |  |
| MV | 10 | | AIC | | 3 | | 0.9649 | | 0.8182 | | 0.6471 | | 1.0000 | | 1.0000 | | 0.6471 | | 0.9298 | | 0.6364 | | 0.6471 | | 0.8972 | | 0.5207 | | 0.6471 | | 0.6835 | |  |
| MV | 15 | | AIC | | 3 | | 0.9912 | | 0.7619 | | 0.7353 | | 0.9825 | | 0.6364 | | 0.7059 | | 1.0000 | | 0.8182 | | 0.7647 | | 0.9738 | | 0.5950 | | 0.6920 | | 0.7612 | |  |
| SV | 5 | | Error | | 11 | | 1.0000 | | 0.8182 | | 0.7059 | | 1.0000 | | 0.8182 | | 0.7059 | | 1.0000 | | 0.8182 | | 0.7059 | | 1.0000 | | 0.8182 | | 0.7059 | | 0.9045 | |  |
| SV | 10 | | Error | | 8 | | 1.0000 | | 0.9091 | | 0.7059 | | 1.0000 | | 0.9091 | | 0.7059 | | 1.0000 | | 0.9091 | | 0.7059 | | 1.0000 | | 0.9091 | | 0.7059 | | 0.9535 | |  |
| SV | 15 | | Error | | 6 | | 1.0000 | | 0.8636 | | 0.7647 | | 1.0000 | | 0.9091 | | 0.7647 | | 1.0000 | | 0.8182 | | 0.7647 | | 1.0000 | | 0.7851 | | 0.7647 | | 0.8861 | |  |
| SV | 5 | | AIC | | 2 | | 0.9912 | | 0.7727 | | 0.7941 | | 0.9825 | | 0.9091 | | 0.8235 | | 1.0000 | | 0.6364 | | 0.7647 | | 0.9738 | | 0.5620 | | 0.7474 | | 0.7398 | |  |
| SV | 10 | | AIC | | 2 | | 0.9912 | | 0.8182 | | 0.7059 | | 1.0000 | | 0.9091 | | 0.7059 | | 0.9825 | | 0.7273 | | 0.7059 | | 0.9738 | | 0.6694 | | 0.7059 | | 0.8074 | |  |
| SV | 15 | | AIC | | 1 | | 1.0000 | | 0.6818 | | 0.6471 | | 1.0000 | | 0.7273 | | 0.6471 | | 1.0000 | | 0.6364 | | 0.6471 | | 1.0000 | | 0.6198 | | 0.6471 | | 0.7873 | |  |
| Mean |  | |  | | 5.83 | | 0.9912 | | 0.8092 | | 0.7059 | | 0.9942 | | 0.8485 | | 0.7059 | | 0.9883 | | 0.7576 | | 0.7059 | | 0.9769 | | 0.7073 | | 0.6984 | | 0.8289 | |  |
| **SNU-1** | | | | | | | | | | | | | | | | | | | | | | | | | | | | | | | | |  |
| MV | 5 | | Error | | 7 | | 0.9877 | | 1.0000 | | 0.6296 | | 0.9697 | | 1.0000 | | 0.6364 | | 1.0000 | | 1.0000 | | 0.6250 | | 0.9550 | | 1.0000 | | 0.6235 | | 0.9772 | |  |
| MV | 10 | | Error | | 7 | | 0.9877 | | 0.8846 | | 0.6296 | | 0.9697 | | 1.0000 | | 0.6364 | | 1.0000 | | 0.8125 | | 0.6250 | | 0.9550 | | 0.7363 | | 0.6235 | | 0.8386 | |  |
| MV | 15 | | Error | | 9 | | 0.9877 | | 0.9231 | | 0.6667 | | 0.9697 | | 0.9000 | | 0.6364 | | 1.0000 | | 0.9375 | | 0.6875 | | 0.9550 | | 0.8843 | | 0.6281 | | 0.9190 | |  |
| MV | 5 | | AIC | | 7 | | 0.9383 | | 0.8462 | | 0.6923 | | 0.8485 | | 0.7000 | | 0.6364 | | 1.0000 | | 0.9375 | | 0.6875 | | 0.7842 | | 0.6243 | | 0.6281 | | 0.6997 | |  |
| MV | 10 | | AIC | | 5 | | 0.9877 | | 0.8462 | | 0.7407 | | 0.9697 | | 0.7000 | | 0.7273 | | 1.0000 | | 0.9375 | | 0.7500 | | 0.9550 | | 0.6243 | | 0.7218 | | 0.7721 | |  |
| MV | 15 | | AIC | | 7 | | 0.9877 | | 0.9231 | | 0.7407 | | 0.9697 | | 0.8000 | | 0.7273 | | 1.0000 | | 1.0000 | | 0.7500 | | 0.9550 | | 0.7200 | | 0.7218 | | 0.8292 | |  |
| SV | 5 | | Error | | 3 | | 0.9877 | | 0.6538 | | 0.6667 | | 0.9697 | | 0.4000 | | 0.6364 | | 1.0000 | | 0.8125 | | 0.6875 | | 0.9550 | | 0.3562 | | 0.6281 | | 0.5832 | |  |
| SV | 10 | | Error | | 10 | | 0.9877 | | 0.8077 | | 0.5556 | | 0.9697 | | 0.9000 | | 0.5455 | | 1.0000 | | 0.7500 | | 0.5625 | | 0.9550 | | 0.7013 | | 0.5445 | | 0.8184 | |  |
| SV | 15 | | Error | | 8 | | 0.9877 | | 0.9231 | | 0.6667 | | 0.9697 | | 1.0000 | | 0.6364 | | 1.0000 | | 0.8750 | | 0.6875 | | 0.9550 | | 0.8203 | | 0.6281 | | 0.8851 | |  |
| SV | 5 | | AIC | | 5 | | 0.9877 | | 0.9615 | | 0.5556 | | 0.9697 | | 0.9000 | | 0.5455 | | 1.0000 | | 1.0000 | | 0.5625 | | 0.9550 | | 0.8550 | | 0.5445 | | 0.9036 | |  |
| SV | 10 | | AIC | | 3 | | 0.9877 | | 0.6154 | | 0.5926 | | 0.9697 | | 0.5000 | | 0.6364 | | 1.0000 | | 0.6875 | | 0.5625 | | 0.9550 | | 0.4824 | | 0.5552 | | 0.6788 | |  |
| SV | 15 | | AIC | | 4 | | 0.9877 | | 0.8077 | | 0.5556 | | 0.9697 | | 0.8000 | | 0.5455 | | 1.0000 | | 0.8125 | | 0.5625 | | 0.9550 | | 0.7962 | | 0.5445 | | 0.8720 | |  |
| Mean |  | |  | | 6.25 | | 0.9835 | | 0.8494 | | 0.6410 | | 0.9596 | | 0.8000 | | 0.6288 | | 1.0000 | | 0.8802 | | 0.6458 | | 0.9408 | | 0.7167 | | 0.6160 | | 0.8147 | |  |
| ^(a)^ Training data set. ^(b)^ Selection data set. ^(c)^ External data set. ^(d)^ Geometric mean of the BCR metric across training and selection sets  ^(e)^ Aggregation algorithm. MV: Majority Vote, SV: Scores Vote. The best performing model per endpoint is highlighted gray | | | | | | | | | | | | | | | | | | | | | | | | | | | | | | | | |  |
