# Supplementary material for: A desirability-based multi objective approach for the virtual screening discovery of broad-spectrum anti-gastric cancer agents
Source: PLoS One. 2018 Feb 8;13(2):e0192176. doi: 10.1371/journal.pone.0192176 (PMC5805264; doi:10.1371/journal.pone.0192176)
Supplement: S2 Table — (DOCX) [file pone.0192176.s002.docx]

# **S2 Table. Mean performance statistics for the base models included in each ensemble**

|  | |  | |  | |  | | Accuracy | | | | | | Sensitivity | | | | | | Specificity | | | | | |
| --- | --- | --- | --- | --- | --- | --- | --- | --- | --- | --- | --- | --- | --- | --- | --- | --- | --- | --- | --- | --- | --- | --- | --- | --- | --- |
| Aggregation^(e)^ | | Max. Models Initial Population | | Objective Function | | Size | | Train^(a)^ | | Sel. ^(b)^ | | Ext. ^(c)^ | | Train^(a)^ | | Sel. ^(b)^ | | Ext. ^(c)^ | | Train^(a)^ | | Sel. ^(b)^ | | Ext.^(c)^ |  |
| **AGS** | | | | | | | | | | | | | | | | | | | | | | | | | |
| MV | 5 | | Error | | 5 | | 0.9651 | | 0.8303 | | 0.7387 | | 0.9294 | | 0.8000 | | 0.7438 | | 0.9885 | | 0.8526 | | 0.7353 | | |
| MV | 10 | | Error | | 7 | | 0.9492 | | 0.8027 | | 0.7258 | | 0.9025 | | 0.7561 | | 0.7100 | | 0.9797 | | 0.8367 | | 0.7361 | | |
| MV | 15 | | Error | | 5 | | 0.9586 | | 0.8303 | | 0.7361 | | 0.9176 | | 0.7951 | | 0.7275 | | 0.9854 | | 0.8564 | | 0.7417 | | |
| MV | 5 | | AIC | | 5 | | 0.9302 | | 0.8096 | | 0.7261 | | 0.8612 | | 0.7610 | | 0.6874 | | 0.9754 | | 0.8455 | | 0.7504 | | |
| MV | 10 | | AIC | | 5 | | 0.9433 | | 0.8121 | | 0.7388 | | 0.8847 | | 0.7610 | | 0.7210 | | 0.9815 | | 0.8495 | | 0.7496 | | |
| MV | 15 | | AIC | | 3 | | 0.9287 | | 0.7796 | | 0.7351 | | 0.8451 | | 0.6992 | | 0.6962 | | 0.9833 | | 0.8385 | | 0.7599 | | |
| SV | 5 | | Error | | 4 | | 0.9186 | | 0.8269 | | 0.7197 | | 0.8397 | | 0.7561 | | 0.6672 | | 0.9702 | | 0.8789 | | 0.7533 | | |
| SV | 10 | | Error | | 3 | | 0.9698 | | 0.8385 | | 0.7169 | | 0.9412 | | 0.8374 | | 0.7131 | | 0.9885 | | 0.8393 | | 0.7195 | | |
| SV | 15 | | Error | | 3 | | 0.9682 | | 0.8179 | | 0.7409 | | 0.9333 | | 0.8130 | | 0.7463 | | 0.9910 | | 0.8214 | | 0.7375 | | |
| SV | 5 | | AIC | | 5 | | 0.9563 | | 0.8227 | | 0.7351 | | 0.9129 | | 0.7951 | | 0.7195 | | 0.9846 | | 0.8429 | | 0.7455 | | |
| SV | 10 | | AIC | | 2 | | 0.9372 | | 0.8082 | | 0.7182 | | 0.8706 | | 0.8049 | | 0.6841 | | 0.9808 | | 0.8106 | | 0.7407 | | |
| SV | 15 | | AIC | | 5 | | 0.9349 | | 0.7828 | | 0.7440 | | 0.8647 | | 0.7512 | | 0.6921 | | 0.9808 | | 0.8058 | | 0.7779 | | |
| Mean |  | |  | | 4.33 | | 0.9467 | | 0.8135 | | 0.7313 | | 0.8919 | | 0.7775 | | 0.7090 | | 0.9825 | | 0.8398 | | 0.7456 | | |
| **NCI-N87** | | | | | | | | | | | | | | | | | | | | | | | | | |
| MV | 5 | | Error | | 5 | | 0.9175 | | 0.7589 | | 0.5499 | | 0.9088 | | 0.8000 | | 0.5412 | | 0.9263 | | 0.7127 | | 0.5581 | | |
| MV | 10 | | Error | | 19 | | 0.9381 | | 0.7585 | | 0.5621 | | 0.9160 | | 0.8029 | | 0.5606 | | 0.9603 | | 0.7129 | | 0.5637 | | |
| MV | 15 | | Error | | 7 | | 0.9687 | | 0.7660 | | 0.5914 | | 0.9524 | | 0.7677 | | 0.5678 | | 0.9850 | | 0.7610 | | 0.6134 | | |
| MV | 5 | | AIC | | 3 | | 0.9678 | | 0.7424 | | 0.6471 | | 0.9708 | | 0.7576 | | 0.6471 | | 0.9649 | | 0.7273 | | 0.6471 | | |
| MV | 10 | | AIC | | 3 | | 0.8830 | | 0.7547 | | 0.6233 | | 0.9240 | | 0.9030 | | 0.5980 | | 0.8421 | | 0.6061 | | 0.6471 | | |
| MV | 15 | | AIC | | 3 | | 0.9591 | | 0.7318 | | 0.6471 | | 0.9240 | | 0.7003 | | 0.6667 | | 0.9942 | | 0.7576 | | 0.6275 | | |
| SV | 5 | | Error | | 11 | | 0.9593 | | 0.7320 | | 0.5811 | | 0.9697 | | 0.7901 | | 0.6237 | | 0.9490 | | 0.6760 | | 0.5375 | | |
| SV | 10 | | Error | | 8 | | 0.9331 | | 0.7470 | | 0.5890 | | 0.9320 | | 0.8136 | | 0.6034 | | 0.9342 | | 0.6818 | | 0.5735 | | |
| SV | 15 | | Error | | 6 | | 0.9006 | | 0.7486 | | 0.6128 | | 0.9298 | | 0.8167 | | 0.6152 | | 0.8713 | | 0.6818 | | 0.6115 | | |
| SV | 5 | | AIC | | 2 | | 0.9430 | | 0.6886 | | 0.6711 | | 0.9298 | | 0.8535 | | 0.6765 | | 0.9561 | | 0.5455 | | 0.6618 | | |
| SV | 10 | | AIC | | 2 | | 0.8728 | | 0.7955 | | 0.6123 | | 0.9298 | | 0.9545 | | 0.7353 | | 0.8158 | | 0.6364 | | 0.4853 | | |
| SV | 15 | | AIC | | 1 | | 1.0000 | | 0.6818 | | 0.6471 | | 1.0000 | | 0.7273 | | 0.6471 | | 1.0000 | | 0.6364 | | 0.6471 | | |
| Mean |  | |  | | 5.83 | | 0.9369 | | 0.7421 | | 0.6112 | | 0.9406 | | 0.8073 | | 0.6235 | | 0.9333 | | 0.6780 | | 0.5978 | | |
| **SNU-1** | | | | | | | | | | | | | | | | | | | | | | | | | |
| MV | 5 | | Error | | 7 | | 0.9471 | | 0.7701 | | 0.5112 | | 0.8831 | | 0.6698 | | 0.4416 | | 0.9911 | | 0.8280 | | 0.5607 | | |
| MV | 10 | | Error | | 7 | | 0.9718 | | 0.7169 | | 0.5334 | | 0.9610 | | 0.7048 | | 0.4948 | | 0.9792 | | 0.7232 | | 0.5601 | | |
| MV | 15 | | Error | | 9 | | 0.9342 | | 0.7262 | | 0.5677 | | 0.8889 | | 0.6975 | | 0.5253 | | 0.9653 | | 0.7407 | | 0.5977 | | |
| MV | 5 | | AIC | | 7 | | 0.8854 | | 0.7116 | | 0.5714 | | 0.7619 | | 0.5778 | | 0.4714 | | 0.9702 | | 0.7923 | | 0.6369 | | |
| MV | 10 | | AIC | | 5 | | 0.9580 | | 0.7289 | | 0.5718 | | 0.9091 | | 0.6356 | | 0.4909 | | 0.9917 | | 0.7875 | | 0.6292 | | |
| MV | 15 | | AIC | | 7 | | 0.9048 | | 0.7101 | | 0.5590 | | 0.7792 | | 0.6111 | | 0.4675 | | 0.9911 | | 0.7720 | | 0.6208 | | |
| SV | 5 | | Error | | 3 | | 0.9877 | | 0.6754 | | 0.6011 | | 0.9697 | | 0.4333 | | 0.5939 | | 1.0000 | | 0.8306 | | 0.6042 | | |
| SV | 10 | | Error | | 10 | | 0.8914 | | 0.7288 | | 0.5363 | | 0.7879 | | 0.6556 | | 0.4182 | | 0.9625 | | 0.7750 | | 0.6204 | | |
| SV | 15 | | Error | | 8 | | 0.8256 | | 0.6923 | | 0.5632 | | 0.7008 | | 0.5875 | | 0.4205 | | 0.9115 | | 0.7578 | | 0.6609 | | |
| SV | 5 | | AIC | | 5 | | 0.9605 | | 0.7292 | | 0.5345 | | 0.9152 | | 0.6600 | | 0.4582 | | 0.9917 | | 0.7717 | | 0.5858 | | |
| SV | 10 | | AIC | | 3 | | 0.9342 | | 0.6841 | | 0.5714 | | 0.8788 | | 0.5519 | | 0.5303 | | 0.9722 | | 0.7653 | | 0.5986 | | |
| SV | 15 | | AIC | | 4 | | 0.9722 | | 0.7019 | | 0.5050 | | 0.9545 | | 0.6250 | | 0.4091 | | 0.9844 | | 0.7500 | | 0.5708 | | |
| Mean |  | |  | | 6.25 | | 0.9311 | | 0.7146 | | 0.5522 | | 0.8658 | | 0.6175 | | 0.4768 | | 0.9759 | | 0.7745 | | 0.6039 | | |
| ^(a)^ Training data set. ^(b)^ Selection data set. ^(c)^ External data set. ^(d)^ Geometric mean of the BCR metric across training and selection sets  ^(e)^ Aggregation algorithm. MV: Majority Vote, SV: Scores Vote. The best performing model per endpoint is highlighted gray | | | | | | | | | | | | | | | | | | | | | | | | | |
