# Supplementary material for: A desirability-based multi objective approach for the virtual screening discovery of broad-spectrum anti-gastric cancer agents
Source: PLoS One. 2018 Feb 8;13(2):e0192176. doi: 10.1371/journal.pone.0192176 (PMC5805264; doi:10.1371/journal.pone.0192176)
Supplement: S3 Table — (DOCX) [file pone.0192176.s003.docx]

# **S3 Table. Parameters for the LS-SVM base models included in VS protocol 10**

| Base Model | Features Subset Index^(a)^ | γ | σ^2^ |
| --- | --- | --- | --- |
| **AGS** | | | |
| 1 | 112, 50, 37, 243, 207, 235 | 15.7125087 | 0.0004949 |
| 2 | 155, 58, 73, 8, 157, 26, 6, 177, 233, 249, 66, 30, 112, 201, 231, 92, 17, 34, 158, 234 | 8403.2643575 | 0.0155333 |
| 3 | 139, 101, 5, 69, 199, 90, 229, 60, 133, 162, 142, 3, 54, 32, 19, 123, 48, 143, 166, 222, 86, 180, 118 | 431.7369989 | 0.2012666 |
| 4 | 248, 57, 179, 193, 14, 6, 104, 56, 20, 73, 121, 61 | 785.6361237 | 0.0088249 |
| 5 | 194, 40, 172, 143, 138, 133, 198, 91, 250, 33, 124, 199, 25, 122, 38, 167, 89, 81, 30, 201, 51, 37 | 602.0486674 | 0.0465709 |
| **NCI-N87** | | | |
| 1 | 143, 191, 19, 50, 245, 81, 131 | 0.938570157 | 0.081414002 |
| 2 | 122, 224, 167, 101, 138, 48, 225, 28 | 6908.27673 | 0.040593958 |
| 3 | 176, 123, 195, 177, 107, 126, 184, 220, 108, 232, 193, 73, 187, 198, 118, 44, 92, 194, 218, 137 | 4.602186407 | 0.171491027 |
| 4 | 136, 210, 4, 202, 42, 224, 12, 183, 76, 11, 73, 22, 124, 114, 152, 178, 47, 90, 213, 118, 18, 225, 93, 81, 55 | 5.109890534 | 0.091422453 |
| 5 | 243, 100, 118, 214, 213, 24, 51, 102, 37, 245, 114, 27, 67, 28, 173, 237, 74, 184 | 2.570351121 | 0.139826081 |
| **SNU-1** | | | |
| 1 | 25, 173, 182, 74, 167, 127, 57, 153, 116, 240, 76, 225, 104 | 39.39976199 | 0.020114696 |
| 2 | 165, 121, 146, 129, 160 | 1590.334965 | 0.003080002 |
| 3 | 57, 18, 159, 11, 160, 144 | 0.439052795 | 0.152778445 |
| 4 | 79, 123, 45, 142, 15, 146, 199, 96, 170, 57, 112, 80, 182, 88, 68 | 19595.56414 | 0.056028542 |
| 5 | 190, 139, 135, 30, 170, 169, 59, 193, 217 | 211.5038209 | 1179.909461 |
| 6 | 101, 128, 225, 112, 214, 116, 205, 143, 104, 70, 98, 88, 45, 107, 126, 177, 165, 64, 140, 239, 127, 110, 59, 189, 92 | 18.56743552 | 0.366648477 |
| 7 | 22, 31, 162, 240, 179, 208, 231, 140, 122, 245, 201, 136, 37, 105, 101, 163, 42, 206, 68, 144, 226, 97 | 155.1382399 | 147.9970285 |
| 8 | 122, 12, 89, 74, 213, 40, 53 | 8.175577327 | 0.009203833 |
| 9 | 133, 102, 11, 121, 129, 168, 107, 25 | 15.29924383 | 0.003873285 |
| 10 | 15, 3, 230, 161, 220, 84, 10, 225, 131, 232, 241, 112, 174, 88, 35, 62 | 6554.919324 | 0.826959422 |
| ^(a)^ The indexes of the features included in each base model correspond to the order the molecular descriptors appear in the HDR files provided as Supporting Information | | | |
